# Evaluation of the Effect of Surgical Extraction of an Impacted Mandibular Third Molar on the Periodontal Status of the Second Molar—Prospective Study

**DOI:** 10.3390/jcm10122655

**Published:** 2021-06-16

**Authors:** Magda Aniko-Włodarczyk, Aleksandra Jaroń, Olga Preuss, Anna Grzywacz, Grzegorz Trybek

**Affiliations:** 1 Department of Oral Surgery, Pomeranian Medical University in Szczecin, 72 Powstańców Wlkp. St., 70-111 Szczecin, Poland; dominika.wlodarczyk@pum.edu.pl (M.A.-W.); jaronola@gmail.com (A.J.); olga.preuss@pum.edu.pl (O.P.); 2 Independent Laboratory of Health Promotion, Pomeranian Medical University in Szczecin, 11 Chlapowskiego St., 70-204 Szczecin, Poland; anna.grzywacz@pum.edu.pl

**Keywords:** third molar, mandibular third molar, impaction, periodontal status, complications

## Abstract

Dental injury to the second molar (SM) caused by the surgical extraction of the impacted third molar tends to be underestimated. The necessity of assessment of the impact of the removal of the wisdom tooth in the mandible on the second molar arose. The study group (*n* = 60) was the one with the second molar on the surgical side, and the control group (*n* = 60) was the one with the tooth on the opposite side of the alveolar arch. Before the surgery, the difficulty level was assessed according to the Pederson scale. The periodontal status of the SM was assessed by probing depth (PD), gingival index (GI), tooth mobility (TM) examination by the percussion method and resonance frequency. Measurements were taken before and after the surgery, 7 days and 8 weeks after the surgery. The study demonstrated the significant impact of the surgical removal of the wisdom tooth on the PD, GI and TM of the SM. The predicted degree of difficulty of the very difficult surgery had an influence on the increase in PD on the distal buccal and lingual surface of the SM, and on the GI in the proximity of the examined tooth. The results of the presented research confirm the necessity of the clinical assessment of the lower SM before and after the surgical removal of the impacted wisdom tooth in the mandible.

## 1. Introduction

The presence of a partially or completely impacted wisdom tooth can cause a deepening of the gingival sulcus and periodontal changes in the distal region of the mandibular second molar [1]. These changes may be asymptomatic and only involve deepening of the gingival sulcus—in addition, there may be redness or a tendency to bleed [2]. A change in the periodontal status of a mandibular second molar may also be a consequence of surgical intervention—this occurs only after surgical removal of an impacted wisdom tooth [3]. The removal of an impacted wisdom tooth carries the risk of traumatizing the second molar, causing it to become more mobile or dislocated during the anteroposterior extraction movements during the procedure. The pressure force generated by the operator using an elevator on the second molar during the removal of the impacted tooth is equal to the resistance that this tooth presents during the final phase of extraction. Despite the frequent coverage of complications associated with surgical removal of wisdom teeth in the scientific literature, increased mobility of the second molar, which can affect the clinical status of the pulp, is often downplayed or overlooked [4]. Clinical and population-based data on the periodontal pathophysiology of the third molar are limited. Information is not collected or even excluded from studies due to the high variability in the morphology and physiology of the wisdom tooth concerning teeth located in the anterior segment of the dental arch. The unclear periodontal status is reflected in the periodontal status of the adjacent tooth, which is the second molar in the mandible. Surgical removal of an impacted mandibular third molar involves soft tissue incision, full-thickness flap dehiscence, alveotomy, or separation, which may adversely affect the periodontal tissues adjacent to the surgical area of the second molar. The data in the literature are not consistent. Surgical intervention on the distal surface of the second lower molar may result in bone loss, periodontal pocket development, or root cement exposure [5,6]. However, some studies confirm an improvement in the level of connective tissue attachment and a reduction in probing depth [7,8]. The analysis of the impact of surgery can be based on the clinical assessment of the pocket depth (PD) and gingival index (GI). The complications of surgical extraction of impacted wisdom teeth in the form of ligamentous damage to the second molar and impaired blood supply to the pulp, which can lead to pulp necrosis, have been overlooked in the literature. In particular, there are no data on the effect of the surgical procedure on changes in the threshold of excitability of the pulp of the second molar, and, thus, its clinical status.

This study aimed to:

1. Evaluate the effect of surgical removal of an impacted third molar on:
  - Clinical probing depth of the mandibular second molar.
  - Gingival condition of the mandibular second molar.
  - Mobility of the mandibular second molar.
2. To determine if there is a relationship between the degree of difficulty of surgical removal of an impacted wisdom tooth and the postoperative probing depth, gingival condition, and mobility of the mandibular second molar.

## 2. Materials and Methods

The study was conducted after obtaining consent from the Bioethics Committee with the number KB-0012/89/16. 

The study included 60 consecutive patients with indications for surgical removal of the third mandibular molar. Adult patients, generally healthy, not taking any permanent medication, and non-smokers, who declared their willingness to participate in the study, were eligible for the procedure. The exclusion criteria were the absence of a second and third mandibular molar in the operated quadrant and on the opposite side in the mandible, the presence of a fixed orthodontic appliance, malocclusions such as distocclusion, mesiocclusion, crowding of teeth, scissor bite, crossbite, tobacco smoking and age below 18 years, and an implanted cardiac electro stimulator. In each patient, the second lower molars were examined—a total of 120 teeth in all patients. We identified two groups:

1. Study group (*n* = 60)—second and third molars in the mandible in the operated quadrant;
2. Control group (*n* = 60)—mandibular second and third molars on the side opposite to the operated side.

Before the study, patients were informed about the study. All participants gave their informed consent to participate in the study and confirmed it with their signature on the form. All patients had a pantographic X-ray taken before the surgery. The degree of impaction was assessed according to Winter [9] and Pell and Gregory [10]. The degree of difficulty of surgical removal of impacted third molars in the mandible was assessed using the Pederson index. All mandibular 2nd molars were vital before surgery, with no prosthetic restoration, only small fillings, and no fractures.

All procedures were performed by three oral surgery specialists with a similar (extensive) level of surgical experience. Tooth extraction was performed under local anesthesia: block anesthesia and infiltration anesthesia 2% with norepinephrine 0.00125% in the amount of 4–6 mL. The first incision was performed with a #15 scalpel at the top of the mandibular alveolar region behind the second molar, and the second releasing incision was performed in the oral vestibule in the distal third of the crown of the second molar. The full-thickness flap was deflected with Molt’s elevator to the level of the external oblique line and stabilized with Langenbeck’s long retractor, resting it on the bone at a ninety-degree angle. With the use of a rubella drill and/or a Lindemann bur, mounted on a surgical handpiece and cooled with a sterile 0.9% sodium chloride solution, the bone was removed from the impacted molar to the level of its neck. Depending on the angle of inclination, the tooth was cut with a drill, and, using elevators and/or forceps, the tooth was removed in whole or in parts. The last stage of the surgical procedure was the wound toilet, which consisted of the removal of bone resulting from the drill cutting, removal of the tooth follicle, possibly inflammatory tissue of granuloma, smoothing of the sharp bone edges, and copious rinsing with a saline solution. The mucoperiosteal flap was then repositioned and stabilized with single knotted sutures using 3-0 silk sutures and left in place for seven days. After surgery, compression with a sterile gauze tampon was applied for 20 min. Patients were advised to maintain postoperative wound hygiene. Patients brushed their teeth after each meal, used a 0.1% chlorhexidine-based rinse and were advised to use nonsteroidal anti-inflammatory drugs in the form of ketoprofen 100 mg twice daily.

### 2.1. Clinical Examination

Clinical examination of the mandibular second molars in both the study and control groups was performed immediately before surgery, seven days after surgery, and eight weeks after surgery. The following parameters were used for clinical evaluation: gingival index, probing depth, impaction, and resonance frequency mobility measurement.

#### 2.1.1. Gingival Assessment

The evaluation was performed using the gingival index (GI) according to Loe and Silness [11]. The index was assessed using a periodontal probe at four measurement points: mesially, distally, vestibularly, and lingually.

#### 2.1.2. Probing Depth (PD) Measurement

The measurement was carried out using a Williams periodontal probe calibrated every 1 mm with an accuracy of 0.5 mm at six measurement points of each second molar—distally, centrally, and mesially on the buccal side and similarly on the lingual side. The probe was inserted into the gingival crevice until gentle resistance, parallel to the long axis of the tooth, and the measurement obtained was archived. 

#### 2.1.3. Tooth Mobility Measurement

The mobility of the mandibular second molars was measured using the Periotest M (Medizintechnik Gulden, Bensheim, Germany) and Osstell (Osstell, Gothenburg, Sweden). In addition to the time sequence appropriate for all other clinical parameters (seven days after surgery and eight weeks after surgery), mobility was also measured immediately after surgery.

#### 2.1.4. Measurement with Periotest M

The mobility was measured on the buccal surface of the second molar based on the percussion method. The head of the device was applied perpendicularly to the buccal surface of the tested tooth at a distance of approximately 2 mm. Correct orientation of the head was indicated by a low tone. To obtain repeatable measurements, the Periotest M was always positioned in the same way in relation to the tooth under test. The test was performed twice for each tooth of the test and control groups. The results expressed on the PTV (Periotest value) scale, displayed on the instrument panel, were recorded in a prepared sheet—the mean value from the two measurements was used in the statistical analysis.

#### 2.1.5. Measurement Using Osstell

The measurement was performed based on resonance frequency analysis. With the mouth wide open, a magnetic sensor (SmartPeg; Osstell, Gothenburg, Sweden) was attached using a composite cured with a polymerization lamp to the chewing surface of the tooth from the test and control groups. The pulse probe was brought approximately one millimeter parallel and perpendicular to the dental arch. The magnetic sensor, along with the polymerized composite, was then removed using light pressure. The readings expressed on the ISQ scale, archived in the memory of the device, were transferred to the developed test card, where their mean value was recorded.

### 2.2. Methodology of Statistical Analysis

Statistical analysis was performed using the statistical package R—version 3.4.2 (R Foundation for Statistical Computing: Vienna, Austria). Qualitative variables were described by the number and percentage of occurrences of each value. Standard measures of position and measures of variability were used to describe quantitative variables. Arithmetic means, standard deviation, median, quartiles, and minimum and maximum values were calculated. 

Qualitative variables that did not have a normal distribution were compared using the Kruskal–Wallis test. The chi-square test was used to compare qualitative variables in the study and control groups. To make a more accurate comparison between the groups, the method of multiple comparisons, i.e., post-hoc analysis (Dunn’s test), was used. 

In the case of small expected values, the Fisher’s exact test was used. The analysis was used to evaluate the effect of the anticipated difficulty of the procedure on the clinical status of the second molar. Quantitative variables were analyzed using the Wilcoxon paired *t*-test and Student’s *t*-test. These were used in the comparison of the clinical status of the 2nd molar from the test and control groups and in the comparative analysis of individual parameters of the clinical status of second molars from the test group between time points. Sequential analysis was used to interpret the change in the clinical status of the second molar teeth between time intervals. 

A value of 0.05 was taken as the level of significance (*p*). All *p* values that were below 0.05 were interpreted as indicating significant relationships. 

## 3. Results

### 3.1. Characteristics of the Study Group

Sixty patients, consecutively presenting for surgical removal of impacted wisdom teeth in the mandible, were included in the clinical study. Among them, there were 17 males and 34 women. Table 1 summarizes the detailed results for the study sample. A total of 60 surgical removals of impacted mandibular third molars were performed with a mean angle of 63.47 degrees (29.34) to the occlusal plane. The spatial position was determined by the position of the wisdom tooth relative to the second molar and mandibular branches. Table 2 shows the detailed characteristics of the position of the third molars and the expected difficulty of the procedure. 

### 3.2. Comparative Analysis of Clinical Status Parameters of Second Molars of the Study and Control Groups before the Procedure, after the Procedure, Seven Days after the Procedure, and Eight Weeks after the Procedure

#### 3.2.1. Comparative Analysis of the Gingival Index (GI) at Second Molars of the Study and Control Groups

Analyses of the GI were performed before surgical removal of the mandibular wisdom tooth, seven days after surgery, and eight weeks after surgical intervention. The mean GI value in the study group before surgery was 0.69 (±0.47), seven days after surgery was 1.65 (±0.47), and eight weeks after surgery was 0.28 (±0.47). There was a significant difference in GI values before surgery compared to seven days after surgical intervention (*p* < 0.001). The results are summarized in Table 3.

#### 3.2.2. Comparative Analysis of Probing Depth at Second Molars of the Study and Control Groups

**Measurement of Probing Depth before Treatment**

Before treatment, the pocket depth in the study group was greatest at the distal–buccal surface, averaging 3.67 mm (1.39). Similar values were also recorded in the control group at 3 mm (1.26). There were significant differences in measurements on the mesial–buccal surface (*p* = 0.003) and distal (*p* = 0.002) and central–lingual surfaces (*p* = 0.046). The results of the full analysis are summarized in Table 4.

**Measurement of Probing Depth Seven Days after Treatment**

All probing depth values seven days after surgery were significantly greater in the study group compared to the control group (*p* < 0.05). As preoperatively, the highest scores in the study and control groups were recorded on the distal–buccal surface with mean measurements of 7.68 mm (2.44) in the study group and 3.13 (1.29) in the control group. The results of the analysis are summarized in Table 5.

**Probing Depth Measurement Eight Weeks after Treatment**

The greatest probing depth eight weeks after treatment was 6 mm distally buccally and lingually in the study group, while the lowest depth was 0.5 mm. Statistical analysis revealed a significant difference in probing depth measurements performed buccally mesially and centrally and lingually mesially, centrally, and distally at second molars between the study and control groups (*p* < 0.05). Eight weeks after the procedure, the mean probing depth in both groups was still greatest on the distal buccal surface. The data are summarized in Table 6.

#### 3.2.3. Comparative Analysis of the Mobility of Second Molars of the Study Group and the Control Group before the Procedure, Seven Days after the Procedure, and Eight Weeks after the Procedure

**Measurement with Periotest M**

The differences in the seventh tooth mobility seven days after treatment in the study and control groups were significant (*p* < 0.001). The study group had higher values averaging 2.27 (−2.55–6.2). No statistically significant differences were observed at the other time points. Preoperatively, and eight weeks postoperatively, both the seventh teeth of the study and control groups were not significantly different in terms of mobility. The remaining results of the statistical analysis are summarized in Table 7.

**Osstell Measurement**

Similar to the mobility measurements performed with the Periotest—the Osstell—after seven days, mobility was significantly higher in the study group compared to the control group (*p* < 0.001). The mean Osstell readings were 46.47 (10.51) and 55.65 (10.56), respectively. The measurement values in the control group decreased, indicating an increase in the seventh tooth mobility. After eight weeks, no significant differences were observed between the study group and the control group. The remaining values were summarized in Table 8.

#### 3.2.4. Gingival Index (GI)

There was a significant relationship between predicted procedure difficulty and gingival index before surgery and seven days after surgery (*p* < 0.05). The GI in patients who were predicted to have a very difficult procedure was statistically significantly higher before surgery than in patients who were predicted to have a moderate or minor procedure (*p* = 0.01). The rate of gingivitis seven days after surgery was significantly higher in patients with a very difficult procedure than in patients after a slightly difficult procedure (*p* = 0.047). The details of the statistical analysis are summarized in Table 9. 

#### 3.2.5. Probing Depths: Before Surgery, Seven Days after Surgery, and Eight Weeks after Surgery

The clinical probing depths obtained at each time point are presented later in the chapter in paragraph four. Statistical analysis revealed that the probing depth on the distal surface of the second molar before the procedure, measured on both the buccal and lingual sides, was significantly different in patients qualified for the procedure with different degrees of difficulty (*p* < 0.005; Kruskal–Wallis test). Using post-hoc analysis, the above relationship was further described. The probing depth of the distal–buccal side in patients who were scheduled for a very difficult procedure was significantly greater before the procedure than in patients who were scheduled for a slightly difficult procedure (*p* = 0.042). Moreover, patients with an anticipated very difficult procedure had a greater probing depth on the distal surface on the lingual side than those with an anticipated procedure with moderate difficulty (*p* = 0.041). Seven days after the procedure, the highest probing depth measurement of 11.5 mm was recorded distally on the buccal surface where the wisdom tooth removal procedure was characterized as moderately difficult. The lowest preoperative PD value recorded was 0.5 mm. The probing depth eight weeks after the procedure showed no significant relationship with the difficulty of the procedure (*p* > 0.05). The details of the analysis performed are summarized in Table 10.

#### 3.2.6. Second Molar Mobility before the Procedure, after the Procedure, Seven Days after the Procedure, and Eight Weeks after the Procedure

**Periotest M Measurement**

The highest values were recorded seven days after the slightly difficult procedure: mean—1.99 (2.55); maximum—6.2. Before the procedure, the mobility of second molars did not differ significantly in terms of the difficulty of the procedure. The mobility immediately after surgery increased significantly and depended on the anticipated difficulty of surgical removal of the third molar (*p* < 0.05; Kruskal–Wallis Test). Post-hoc analysis showed that, in patients after a slightly difficult procedure, the mobility of the second molar was significantly higher than in patients after a moderately difficult procedure (*p* = 0.043). 

There were no significant differences in second molar mobility according to the difficulty of the procedure at seven days and eight weeks after the procedure. The results are presented in Table 11.

**Osstell Measurement**

Osstell readings, obtained at particular time points, are presented. Statistical analysis showed no significant correlation between the mobility of the seventh tooth measured with the Osstell and the anticipated difficulty of wisdom tooth removal surgery. The highest mean measurement value was 60.3 (8.38) eight weeks after the slightly difficult procedure. The results of the statistical analysis were summarized in Table 12.

## 4. Discussion

Surgical removal of the impacted third molar is a commonly performed procedure by dental surgery specialists. It is estimated that 16.7–73% of the world population presents at least one impacted molar, most commonly in the mandible [12,13,14]. Parafunctions and abnormal eating habits, such as the consumption of soft textured foods by children, lead to abnormal oral development and consequently malocclusion [15,16]. The most common cause of tooth impaction is a deficit of space in the dental arch, impaction can also be the result of an abnormal position and path of eruption of the bud, tooth morphology, or function of the dental follicle, and genetic causes [17,18,19]. Over the last 40 years, there has been an increase in the incidence of tooth impaction, which is a consequence of the development of civilization. It should be predicted that health needs in this area will continue to increase.

Surgical removal of an impacted lower wisdom tooth requires interference into the soft and hard tissues. The procedure involves incision of tissues, often with the performance of vestibular alveotomy, distal–lingual, or crown–root separation of the removed tooth. The surgical removal of wisdom teeth carries a variable risk of complications, and their occurrence depends on factors such as the location of the tooth, the age and general condition of the patient, the difficulty of the procedure, as well as the knowledge and experience of the operator. Complications associated with the surgical removal of wisdom teeth can be divided into those arising during and after the procedure. During the procedure, complications may arise in connection with the impacted or adjacent tooth, soft and hard tissues, inferior alveolar nerve, or lingual nerve. However, postoperatively, pain, swelling, trismus, infection, bleeding, delayed healing, and wound edge dehiscence may occur [20,21]. There are many methods to reduce non-invasive post-surgical complications, which include kinesio taping (KT). KT application is an effective method for reducing postoperative edema, pain, and trismus after impacted mandibular wisdom teeth surgery [22].

There are many papers available in the literature regarding perioperative complications associated with surgical removal of wisdom teeth [20,21,23,24,25,26,27,28]. However, the impact of surgical removal of wisdom teeth in the mandible on the postoperative status of the second lower molar is marginalized or completely ignored. Consequently, no algorithm has been developed to evaluate the clinical status of the second molar after surgery. Thus, there is a need to develop a useful scheme for its monitoring. This would allow us to predict the potential risk of complications associated with perioperative trauma suffered by the second lower molar.

The probing depth (PD) measurement of the mandibular second molar was adapted from a scenario by the team of Faria et al. [29], in which measurements were taken on the distal surface of the tooth buccally and lingually. In addition, in our work, PD measurements on the mentioned surfaces were supplemented by four additional surfaces with the following locations: centrally buccal and lingual and mesially buccal and lingual, examining both second lower molars. The six-surface examination scheme allowed for a more complete assessment of the periodontal status, especially in conjunction with mobility, as PD has a direct impact on it [30]. Authors Chou et al. [31] included patients in the study group of similar age, with a mean of 45.12 years, ranging from 26 to 73 years of age. The study included second molars in 42 patients who underwent surgical removal of wisdom teeth in the mandible. Each tooth was classified into the appropriate group based on its position relative to the occlusal plane. However, the authors did not provide information on the time elapsed since tooth extraction, which was strictly defined in our study. The probing depth measured in the distal part of the buccal surface of the second molar was significantly greater after wisdom tooth removal compared to the tooth on the opposite side of the arch where the procedure was not performed (*p* = 0.004) [31]. Similar correlations, that is, a significant deepening of probing on the distal surface, were obtained in our study seven days after treatment (*p* < 0.001). Our study, in the assumptions evaluating the clinical condition of second molars, was extended to evaluate the probing depth at additional points on the buccal and lingual surfaces of second molars, unlike the study by Tabrizi et al. [32], who probed only the distal part of the tooth. Measurements were taken at three points on the distal surface, arguing that this was the greatest tissue traumatization during the procedure in the mentioned area. The age of the patients was similar to the age in the present study and a mean of 20.9 (18 to 25 years). Forty-two patients who underwent surgical removal of an impacted third molar were included in the study. All teeth were in a mesioangular position and belonged to group C1 according to the Pell and Gregory classification. In our study, a greater variety of teeth qualifying for surgery was observed, with mesial angle teeth accounting for 50%. The intraoperative procedure and the type of flap created were similar to the study by Tabrizi et al. [32]. The authors compared all measurements with the preoperative state, without a control group. A significant increase in pocket depth was observed after 26 weeks compared to the preoperative status (*p* = 0.012). The mean pocket depth before surgery was 2.71 mm (±0.59) [32]. In our study, at a shorter time after eight weeks, a significant reduction in depth was observed in the distal part of the buccal (*p* = 0.007) and lingual (*p* < 0.001) surfaces compared to the pre-treatment condition. Tooth mobility, due to trauma and/or periodontal disease, is defined as the movement of the tooth in the horizontal and/or vertical planes under the influence of forces applied by the examiner [33]. An increase in tooth mobility can be caused by the loss of one of the alveolar bone walls that provides support for the tooth embedded in it [34]. The above situation often occurs after surgical removal of a lower impacted tooth, where the distal bony support of the second molar is lost.

Czechowska et al. [24] described a case of partial dislocation of the second molar—47—during surgical removal of an impacted wisdom tooth in the mandible—48. Radiological analysis revealed horizontal impaction of the third molar and, according to Pell–Gregory, it was classified as group B. Surgical intervention resulted in the subluxation of tooth 47, which required immobilization. The consequence was pulp necrosis and the need for endodontic treatment [24].

There are no data in the literature on what percentage of second molars the ligamentous apparatus weakens. Studies conducted show that there is a transient increase in mobility shortly after surgical removal of wisdom teeth but within the physiological range.

Ye et al. [4], based on the analysis of cone-beam tomography images, performed a preoperative computer simulation of the procedure, adequate to the position and impaction of the lower wisdom tooth. The developed method allowed the successful removal of the impacted wisdom tooth in different degrees of impaction. According to the authors, adequate osteotomy and separation of the tooth can reduce the potential risk of injury to the adjacent tooth. However, despite such careful preoperative diagnosis and individualized surgical planning, the researchers were unable to prevent partial dislocation of the second molar, which is near the operated area. Subluxation occurred in one of 136 mandibular second molars studied. The article does not state how the degree of tooth mobility was assessed (Ye et al., 2016) [4]. Monitoring the mobility of the second molar before and after surgical removal of the wisdom tooth in the mandible allows us to indirectly assess the loss of bone support and the forces that acted on the tooth during the surgical intervention. According to some authors, the increase in tooth mobility caused by surgical intervention has a direct bearing on the magnitude of the pulp excitability threshold tested by the electrical test [35,36,37]. In our study, the last measurements after eight weeks, performed with Osstell and Periotest M, differed. Second molars showed a lower degree of mobility as measured with Osstell than with Periotest M. The method of testing probably underlies this discrepancy. The specificity of Periotest M only allows mobility to be tested in the vestibulo-lingual direction. Osstell, on the other hand, is a composite of vestibulo-lingual and mesiodistal mobility. The results of the present study indicate the need for evaluation of the clinical condition of the second molar before surgery and periodic monitoring after removal of the impacted third molar in the mandible. The evaluation should be based on the study of parameters such as probing depth, mobility, and gingival index. This management algorithm, augmented by Pederson’s degree of difficulty assessment, helps minimize complications associated with the clinical condition of the second molar and is often overlooked in the diagnosis and treatment of complications after surgical removal of an impacted wisdom tooth in the mandible. It is important to emphasize the significant impact of tobacco smoking on oral health, particularly the periodontal status. Therefore, we excluded all smokers from the study [38].

It should be emphasized that this study has limitations. It was short—only an 8-week follow-up—; however, it is conditioned by the healing time of soft tissues (24–35 days) [39] and hard tissues (8 weeks) [40] in the oral cavity. In addition, the study included subjects with varying degrees of retention and difficulty, which may have had different effects on the 2nd molar. Sixty consecutively enrolled patients who met the inclusion and exclusion criteria were included in the study, regardless of the anticipated difficulty of the procedure or degree of retention. Because the control group was the tooth on the opposite side of the mandible, different degrees of retention and difficulty of surgery were considered. Another limitation of the study is the fact that teeth from the opposite quadrant were not included in the study, due to the lack of a control group in this case (because the operator had already removed the third molar on one side of the mandible). It should also be noted that in the literature there are also new scales for assessing the difficulty of the procedure, e.g., taking into account the time of the procedure [41,42].

## 5. Conclusions

The surgical removal of an impacted third molar in the mandible significantly affects the clinical probing depth of the second lower molar, causes a significant increase in the gingivitis index shortly after the procedure, and significantly increases the mobility of the second molar shortly after the procedure. There is a relationship between the degree of difficulty of removal of an impacted third molar in the mandible and the postoperative probing depth and mobility of the second molar and the gingivitis index value. The results of the present study support the need for a clinical evaluation of the second lower molar before and after surgical removal of an impacted wisdom tooth in the mandible.

## Figures and Tables

**Table 1:** Characteristics of the study patients.

		Mean (SD)	Median (Quartil)	IQR
Age		24.82 (5.51)	23 (21–28)	27
		***n***	**(%)**	
Sex	Woman	43	71.67	
	Man	17	28.33	

Explanations: SD—standard deviation; *n*—number of subjects.

**Table 2:** Characteristics of the position of lower wisdom teeth.

		Mean (SD)	Median (Quartil)	IQR
Angultion		63.47 (29.34)	74.45 (40–87.75)	47.75
		***n***	**(%)**	
Winter	Mesioangular	30	50.00	
	Horizontal	7	11.67	
	Vertical	16	26.67	
	Distoangular	7	11.67	
Pell and Gregory	Level A	30	50.00	
	Level B	19	31.67	
	Level C	11	18.33	
Pell and Gregory	Class 1	9	15.00	
	Class 2	40	66.67	
	Class 3	11	18.33	
Difficulty of the procedure (Pederson)	Slightly difficult	10	16.67	
	Moderately difficult	36	60.00	
	Very difficult	14	23.33	

Explanations: SD—standard deviation; *n*—number of patients.

**Table 3:** Comparison of the GI before surgery, 7 days after surgery, and 8 weeks after surgery.

GI	Group	*n*	Mean	SD	Median	Min	Max	Q1	Q3	IQR	*p* *
Before the procedure	Study	60	0.69	0.47	0.5	0	2	0.5	0.81	0.31	0.002
	Control	60	0.5	0.42	0.5	0	2	0.25	0.75	0.5	
After 7 days	Study	60	1.65	0.47	1.75	0.5	2.5	1.5	2	0.5	<0.001
	Control	60	0.62	0.46	0.5	0	2.5	0.25	1	0.75	
After 8 weeks	Study	60	0.28	0.33	0.25	0	1.25	0	0.5	0.5	0.081
	Control	60	0.4	0.43	0.25	0	2.25	0	0.5	0.5	

* Wilcoxon test for dependent (repeated) measurements. Explanations: *n*—number of teeth; SD—standard deviation; Min—minimum value; Max—maximum value; Q1—first quartile; Q3—third quartile; *p*—significance level.

**Table 4:** Comparison of probing depth at the second molar in the study and control groups before treatment.

PD (mm)	Group	*n*	Mean	SD	Median	Min	Max	Q1	Q3	IQR	*p* *
Buccal—m	Study	60	1.52	0.67	1.5	0.5	3.5	1	2	0.5	0.003
	Control	60	1.31	0.58	1	0.5	4	1	1.5	0.5	
Buccal—c	Study	60	1.62	0.69	1.5	0.5	3	1	2	1	0.077
	Control	60	1.47	0.58	1.5	0.5	3	1	2	1	
Buccal—d	Study	60	3.67	1.39	3.5	1	8	3	4.62	1.62	0.002
	Control	60	3	1.26	3	1	5.5	2	3.5	1.5	
Lingual—m	Study	60	1.54	0.61	1.5	0.5	3.5	1	2	1	0.667
	Control	60	1.48	0.68	1	0.5	4	1	2	1	
Lingual—c	Study	60	1.71	0.63	1.5	0.5	3.5	1	2	1	0.046
	Control	60	1.51	0.6	1.5	0.5	3	1	2	1	
Lingual—d	Study	60	3.38	1.21	3.5	1	5.5	2.88	4	1.12	0.16
	Control	60	3.17	1.14	3.5	1	5.5	2	3.5	1.5	

* Wilcoxon test for dependent (repeated) measurements. Notes: *n*—number of teeth; SD—standard deviation; Min—minimum value; Max—maximum value; Q1—first quartile; Q3—third quartile; *p*—significance level; m—mesially; c—centrally; d—distally.

**Table 5:** Comparison of probing depth at the second molar of the study and control groups measured 7 days after treatment.

PD (mm)	Group	*n*	Mean	SD	Median	Min	Max	Q1	Q3	IQR	*p* *
Buccal—m	Study	60	2.08	0.84	2	1	5	1.5	2.5	1	<0.001
	Control	60	1.52	0.74	1	0.5	4	1	2	1	
Buccal—c	Study	60	2.91	1.33	3	1	8	2	3.62	1.62	<0.001
	Control	60	1.57	0.63	1.5	0.5	3.5	1	2	1	
Buccal—d	Study	60	7.68	2.44	8	3	12	5.5	9.62	4.12	<0.001
	Control	60	3.13	1.29	3	1	5.5	2	3.62	1.62	
Lingual—m	Study	60	1.95	0.82	2	0.5	4	1	2	1	0.007
	Control	60	1.62	0.7	1.5	1	4	1	2	1	
Lingual—c	Study	60	2.28	1.01	2	1	5	1.5	3	1.5	<0.001
	Control	60	1.62	0.67	1.5	1	3.5	1	2	1	
Lingual—d	Study	60	4.68	1.78	4	2	10.5	3.5	5.62	2.12	<0.001
	Control	60	3.18	1.12	3	1	5.5	2	4	2	

* Wilcoxon test for dependent (repeated) measurements. Explanations: *n*—number of teeth; SD—standard deviation; Min—minimum value; Max—maximum value; Q1—first quartile; Q3—third quartile; *p*—significance level.

**Table 6:** Comparison of probing depth at the second molar of the study and control groups measured 8 weeks after the procedure.

PD (mm)	Group	*n*	Mean	SD	Median	Min	Max	Q1	Q3	IQR	*p* *
Buccal—m	Study	60	1.69	0.58	2	0.5	3	1	2	1	0.009
	Control	60	1.43	0.71	1	0.5	4	1	2	1	
Buccal—c	Study	60	1.79	0.59	2	1	4	1	2	1	0.001
	Control	60	1.5	0.74	1.25	0.5	5.5	1	2	1	
Buccal—d	Study	60	3.05	0.94	3	1.5	6	2	3.5	1.5	0.202
	Control	60	2.83	1.16	2.5	1	5.5	2	3.5	1.5	
Lingual—m	Study	60	1.73	0.65	2	1	4	1	2	1	0.017
	Control	60	1.47	0.71	1	0.5	4	1	2	1	
Lingual—c	Study	60	1.82	0.73	2	1	4	1	2	1	0.007
	Control	60	1.5	0.62	1.5	0.5	3	1	2	1	
Lingual—d	Study	60	2.62	0.95	2.5	1	6	2	3	1	0.319
	Control	60	2.85	1.14	2.75	1	5.5	2	3.5	1.5	

* Wilcoxon test for dependent (repeated) measurements. Explanations: *n*—number of teeth; SD—standard deviation; Min—minimum value; Max—maximum value; Q1—first quartile; Q3—third quartile; *p*—significance level.

**Table 7:** Comparison of the mobility of second molars of the study and control groups at different time points as measured by Periotest M.

Periotest	Group	*n*	Mean	SD	Median	Min	Max	Q1	Q3	IQR	*p* *
Before the procedure	Study	60	−0.68	2.18	−1	−5.85	5.7	−1.8	0.54	−1.26	0.965
	Control	60	−0.7	1.97	−0.78	−5.35	4.15	−1.8	0.66	−1.14	
After 7 days	Study	60	1.2	2.27	0.95	−2.55	6.2	−0.4	3.01	2.61	<0.001
	Control	60	−0.46	1.85	−0.62	−4.25	4.05	−1.61	0.74	−0.87	
After 8 weeks	Study	60	−0.18	2.46	−0.4	−8	4.9	−1.51	1.27	−0.24	0.096
	Control	60	−0.6	1.82	−0.62	−4.3	3.95	−1.56	0.65	−0.91	

* Wilcoxon test for dependent (repeated) measurements. Explanations: *n*—number of teeth; SD—standard deviation; Min—minimum value; Max—maximum value; Q1—first quartile; Q3—third quartile; *p*—significance level.

**Table 8:** Comparison of mobility of second molars of the study and control groups at different time points as measured by Osstell.

Osstell	Group	*n*	Mean	SD	Median	Min	Max	Q1	Q3	IQR	*p* *
Before the procedure	Study	60	55.26	11.47	54	23.5	84	48.25	64	15.75	0.471
	Control	60	56.28	10.54	59.75	27	73.5	48.38	64.5	16.12	
After 7 days	Study	60	46.47	10.51	48.5	19.5	66.5	39.75	54	14.25	<0.001
	Control	60	55.65	10.56	57.75	27.5	70.5	49.5	64.5	15	
After 8 weeks	Study	60	59.98	9.23	63	34.5	72.5	55.38	66.62	11.24	0.604
	Control	60	58.49	11.86	62.75	−1.3	71.5	52	66.5	14.5	

* Wilcoxon test for dependent (repeated) measurements. Explanations: *n*—number of teeth; SD—standard deviation; Min—minimum value; Max—maximum value; Q1—first quartile; Q3—third quartile; *p*—significance level.

**Table 9:** Comparison of the relationship of predicted procedure difficulty and the gingival index of the second molar before surgery, 7 days after surgery, and 8 weeks after surgery.

GI	Difficulty of the Procedure	*n*	Mean	SD	Median	Min	Max	Q1	Q3	IQR	*p* *
Before the procedure	Slightly difficult	10	0.48	0.14	0.5	0.25	0.75	0.5	0.5	0	0.01
	Moderately difficult	36	0.64	0.49	0.5	0	2	0.44	0.81	0.37	B >
	Very difficult	14	0.98	0.46	0.75	0.5	1.75	0.56	1.5	0.94	U. N
After 7 days	Slightly difficult	10	1.35	0.5	1.25	0.5	2.25	1.06	1.5	0.44	0.047
	Moderately difficult	36	1.68	0.45	1.62	0.5	2.5	1.5	2	0.5	B >
	Very difficult	14	1.79	0.45	2	0.75	2.5	1.75	2	0.25	N
After 8 weeks	Slightly difficult	10	0.2	0.33	0	0	0.75	0	0.38	0.38	0.527
	Moderately difficult	36	0.28	0.33	0.25	0	1.25	0	0.5	0.5	
	Very difficult	14	0.32	0.36	0.25	0	1.25	0	0.5	0.5	

* Kruskal–Wallis test + post-hoc analysis (Dunn’s test). Explanations: *n*—number of teeth; SD—standard deviation; Min—minimum value; Max—maximum value; Q1—first quartile; Q3—third quartile; *p*—significance level; N—slightly difficult procedure; U—moderately difficult procedure; B—very difficult procedure.

**Table 10:** Comparison of the relationship of predicted treatment difficulty and probing depth before treatment, 7 days after treatment, and 8 weeks after treatment.

PD (mm)		Difficulty of the Procedure	*n*	Mean	SD	Median	Min	Max	Q1	Q3	IQR	*p* *
Before the procedure	Buccal—m	Slightly difficult	10	1.2	0.54	1	0.5	2.5	1	1.38	0.38	0.162
		Moderately difficult	36	1.6	0.72	1.5	1	3.5	1	2	1	
		Very difficult	14	1.57	0.58	1.75	0.5	2.5	1	2	1	
	Buccal—c	Slightly difficult	10	1.15	0.53	1	0.5	2	1	1.38	0.38	0.058
		Moderately difficult	36	1.71	0.67	1.75	1	3	1	2	1	
		Very difficult	14	1.71	0.75	1.75	1	3	1	2	1	
	Buccal—d	Slightly difficult	10	3	1.2	3	1	5.5	2.25	3.5	1.25	0.042
		Moderately difficult	36	3.61	1.44	3.5	1	8	3	4	1	B >
		Very difficult	14	4.29	1.16	4.25	2	5.5	3.5	5.5	2	N
	Lingual—m	Slightly difficult	10	1.45	0.86	1	0.5	3.5	1	1.88	0.88	0.445
		Moderately difficult	36	1.6	0.58	1.5	1	3.5	1	2	1	
		Very difficult	14	1.46	0.5	1.5	1	2.5	1	1.88	0.88	
	Lingual—c	Slightly difficult	10	1.75	0.86	1.75	0.5	3.5	1.12	2	0.88	0.237
		Moderately difficult	36	1.78	0.55	2	1	3	1.5	2	0.5	
		Very difficult	14	1.5	0.65	1.25	1	3	1	1.88	0.12	
	Lingual—d	Slightly difficult	10	2.95	1.26	3	1	5.5	2.25	3.5	1.25	0.041
		Moderately difficult	36	3.26	1.17	3.5	1	5.5	2.38	4	1.62	B >
		Very difficult	14	3.96	1.13	4	2	5.5	3.5	4.75	1.25	U
After 7 days	Buccal—m	Slightly difficult	10	2.15	1.08	2	1	5	1.62	2	0.38	0.454
		Moderately difficult	36	2.12	0.74	2	1	3.5	1.88	2.5	0.62	
		Very difficult	14	1.89	0.92	2	1	4	1	2	1	
	Buccal—c	Slightly difficult	10	3.2	2.24	2	1	8	1.62	4.75	3.13	0.964
		Moderately difficult	36	2.86	1.08	3	1	5	2	3.5	1.5	
		Very difficult	14	2.82	1.17	2.75	1	5	2	3	1	
	Buccal—d	Slightly difficult	10	7.35	1.76	8	4	10	6.25	8	1.75	0.075
		Moderately difficult	36	7.26	2.44	7	3	11.5	5	9.5	4.5	
		Very difficult	14	9	2.56	8.5	4	12	8	11	3	
	Lingual—m	Slightly difficult	10	1.55	0.6	1.5	1	2.5	1	2	1	0.068
		Moderately difficult	36	2.17	0.86	2	1	4	1.88	3	1.12	
		Very difficult	14	1.68	0.7	2	0.5	3	1	2	1	
	Lingual—c	Slightly difficult	10	1.8	1.01	1.25	1	3.5	1	2.75	1.75	0.165
		Moderately difficult	36	2.44	1.05	2	1	5	2	3	1	
		Very difficult	14	2.21	0.85	2	1	4	2	2.5	0.5	
	Lingual—d	Slightly difficult	10	4.1	1.43	4	2	6	3.5	5	1.5	0.55
		Moderately difficult	36	4.61	1.46	4.25	2	8	3.88	5.5	1.62	
		Very difficult	14	5.29	2.55	4.5	2	10.5	3.62	6.75	3.13	
After 8 weeks	Buccal—m	Slightly difficult	10	1.7	0.54	2	1	2.5	1.12	2	0.88	0.975
		Moderately difficult	36	1.67	0.46	2	1	2	1	2	1	
		Very difficult	14	1.75	0.85	2	0.5	3	1	2	1	
	Buccal—c	Slightly difficult	10	1.9	0.61	2	1	3	1.62	2	0.38	0.759
		Moderately difficult	36	1.75	0.42	2	1	2	1.5	2	0.5	
		Very difficult	14	1.82	0.91	2	1	4	1	2	1	
	Buccal—d	Slightly difficult	10	2.8	0.98	2.75	1.5	4.5	2	3.38	1.38	0.481
		Moderately difficult	36	3.03	0.86	3	2	5	2.38	3.12	0.74	
		Very difficult	14	3.29	1.12	3.25	2	6	2.25	4	1.75	
	Lingual—m	Slightly difficult	10	1.7	0.54	2	1	2.5	1.12	2	0.88	0.397
		Moderately difficult	36	1.78	0.61	2	1	4	1.38	2	0.62	
		Very difficult	14	1.61	0.81	1.25	1	3.5	1	2	1	
	Lingual—c	Slightly difficult	10	1.9	1.07	1.75	1	4	1	2	1	0.615
		Moderately difficult	36	1.88	0.71	2	1	4	1.5	2	0.5	
		Very difficult	14	1.64	0.46	2	1	2	1.12	2	0.88	
	Lingual—d	Slightly difficult	10	2.9	1.2	2.5	2	5	2	3.25	1.25	0.266
		Moderately difficult	36	2.44	0.75	2	1	5	2	3	1	
		Very difficult	14	2.89	1.15	3	1	6	2.12	3	0.88	

* Kruskal–Wallis test + post-hoc analysis (Dunn’s test). Explanations: *n*—number of teeth; SD—standard deviation; Min—minimum value; Max—maximum value; Q1—first quartile; Q3—third quartile *p*—significance level; m—mesial; c—central; d—distally; N—slightly difficult procedure; U—moderately difficult procedure; B—very difficult procedure.

**Table 11:** Comparison of the relationship of predicted procedure difficulty and second molar mobility before the procedure, immediately after the procedure, 7 days after the procedure, and 8 weeks after the procedure (measured by Periotest M).

Periotest	Difficulty of the Procedure	*n*	Mean	SD	Median	Min	Max	Q1	Q3	IQR	*p* *
Before the procedure	Slightly difficult	10	0.82	2.31	−0.05	−1.55	4.15	−0.99	3.15	2.16	0.091
	Moderately difficult	36	−0.91	2.12	−1.23	−5.85	5.7	−2.06	0.19	−1.87	
	Very difficult	14	−1.15	1.91	−1.1	−5.45	1.4	−1.79	0.14	−1.65	
After the procedure	Slightly difficult	10	1.99	2.55	1.82	−0.55	6.2	−0.49	3.79	3.3	0.044
	Moderately difficult	36	−0.07	1.77	−0.32	−3.4	4.55	−1.42	0.86	−0.56	N >
	Very difficult	14	0.43	2.5	1.1	−5.2	4.6	−0.51	1.85	1.34	U
After 7 days	Slightly difficult	10	1.94	1.87	2.27	−0.75	4.3	0.48	3.09	2.61	0.433
	Moderately difficult	36	1.08	2.38	0.8	−2.55	5.85	−0.85	2.51	3.36	
	Very difficult	14	0.98	2.28	0.85	−2.35	6.2	0.11	1.71	1.6	
After 8 weeks	Slightly difficult	10	0.74	2.24	0.02	−1.65	4.9	−0.87	2.22	1.35	0.529
	Moderately difficult	36	−0.46	2.44	−0.45	−8	4.5	−1.6	1.02	−0.58	
	Very difficult	14	−0.15	2.66	−0.57	−5	4.25	−1.11	1.66	0.55	

* Kruskal–Wallis test + post-hoc analysis (Dunn’s test). Explanations: *n*—number of teeth; SD—standard deviation; Min—minimum value; Max—maximum value; Q1—first quartile; Q3—third quartile; *p*—significance level; N—slightly difficult procedure; U—moderately difficult procedure; B—very difficult procedure.

**Table 12:** Comparison of the relationship of predicted treatment difficulty and second molar mobility before treatment, immediately after treatment, 7 days after treatment, and 8 weeks after treatment (Osstell measurement).

Osstell	Difficulty of the Procedure	*n*	Mean	SD	Median	Min	Max	Q1	Q3	IQR	*p* *
Before the procedure	Slightly difficult	10	58.25	13.48	61.5	33.5	80	57.25	64	6.75	0.394
	Moderately difficult	36	55.75	11.35	53.75	23.5	84	49.25	63.62	14.37	
	Very difficult	14	51.86	10.19	52.5	38.5	67.5	42.5	56.25	13.75	
After the procedure	Slightly difficult	10	48.45	15.48	54.75	24	64	34.38	59.62	25.24	0.135
	Moderately difficult	36	46.94	13.37	49.5	12	66	38	59	21	
	Very difficult	14	40.43	9.81	41.25	24.5	58	32	45.38	13.38	
After 7 days	Slightly difficult	10	48.95	12.57	52	25.5	64.5	43.5	54	10.5	0.624
	Moderately difficult	36	46.4	10.22	47	19.5	66.5	41.5	53.12	11.62	
	Very difficult	14	44.86	10.16	47.75	27.5	57.5	35.25	53.5	18.25	
After 8 weeks	Slightly difficult	10	60.3	8.38	60.75	44	72.5	56	65.62	9.62	0.877
	Moderately difficult	36	59.76	9.97	64.75	34.5	70.5	52.88	67	14.12	
	Very difficult	14	60.29	8.39	63	42.5	72.5	57.12	65.62	8.5	

* Kruskal–Wallis test. Explanations: *n*—number of teeth; SD—standard deviation; Min—minimum value; Max—maximum value; Q1—first quartile; Q3—third quartile; *p*—significance level.

## Data Availability

Data available on request.
